# Excision of an Unstable Pathogenicity Island in *Salmonella enterica* Serovar Enteritidis Is Induced during Infection of Phagocytic Cells

**DOI:** 10.1371/journal.pone.0026031

**Published:** 2011-10-19

**Authors:** Tania S. Quiroz, Pamela A. Nieto, Hugo E. Tobar, Francisco J. Salazar-Echegarai, Rodrigo J. Lizana, Carolina P. Quezada, Carlos A. Santiviago, Daniela V. Araya, Claudia A. Riedel, Alexis M. Kalergis, Susan M. Bueno

**Affiliations:** 1 Departamento de Genética Molecular y Microbiología, Facultad de Ciencias Biológicas, Millennium Institute on Immunology and Immunotherapy, Pontificia Universidad Católica de Chile, Santiago, Chile; 2 Departamento de Bioquímica y Biología Molecular, Facultad de Ciencias Químicas y Farmacéuticas, Universidad de Chile, Santiago, Chile; 3 Facultad de Ciencias Biológicas y Facultad de Medicina, Millennium Institute on Immunology and Immunotherapy, Universidad Andrés Bello, Santiago, Chile; 4 Departamento de Reumatología, Facultad de Medicina, Pontificia Universidad Católica de Chile, Santiago, Chile; East Carolina University School of Medicine, United States of America

## Abstract

The availability of the complete genome sequence of several *Salmonella enterica* serovars has revealed the presence of unstable genetic elements in these bacteria, such as pathogenicity islands and prophages. This is the case of *Salmonella enterica* serovar Enteritidis (*S.* Enteritidis), a bacterium that causes gastroenteritis in humans and systemic infection in mice. The whole genome sequence analysis for *S.* Enteritidis unveiled the presence of several genetic regions that are absent in other *Salmonella* serovars. These regions have been denominated “regions of difference” (ROD). In this study we show that ROD21, one of such regions, behaves as an unstable pathogenicity island. We observed that ROD21 undergoes spontaneous excision by two independent recombination events, either under laboratory growth conditions or during infection of murine cells. Importantly, we also found that one type of excision occurred at higher rates when *S.* Enteritidis was residing inside murine phagocytic cells. These data suggest that ROD21 is an unstable pathogenicity island, whose frequency of excision depends on the environmental conditions found inside phagocytic cells.

## Introduction

*S.* Enteritidis is a Gram negative bacterium and the etiological agent of several foodborne diseases in humans [1]. This bacterium belongs to the subspecies I of the species *Salmonella enterica*, whose members cause systemic diseases in warm-blooded animals [2], [3]. The ability of *S.* Enteritidis to cause a systemic disease in the host is due to its capacity to survive and replicate inside eukaryotic cells, especially within epithelial and phagocytic cells [4]. This feature of *S.* Enteritidis promotes the establishment of systemic disease in mammals and birds after ingestion of contaminated food or water [5].

As for many other Enterobacteria, the complete genome of *S.* Enteritidis has been sequenced and analyzed [2]. That information has allowed the identification of several genetic regions absent in the genome of other *Salmonella* serovars, such as Typhimurium. These distinctive gene clusters, denominated “Regions of difference” (ROD), could have been acquired by means of lateral gene transfer [2]. One of such regions is ROD21, a pathogenicity island found only in the chromosome of *S.* Enteritidis, *S.* Gallinarum and *S.* Dublin, but absent in other *Salmonella* serovars whose whole genome has been sequenced [2], [6]. Similar to other pathogenicity islands described in Enterobacteria, ROD21 is located next to a gene coding for a tRNA. Previous reports have shown that genomic islands of Enterobacteria located near tRNA genes are unstable, because they excise from the bacterial chromosome [7]–[9]. For instance, it has been described that pathogenicity islands SHI-1 and SRL of *Shigella flexneri* excise from the bacterial chromosome in laboratory growth conditions [10], as well as the high pathogenicity island of *Yersinia pseudotuberculosis* [11], [12]. In *Salmonella*, previous reports have shown that SPI-7 of serovar Typhi, a 133 kb genomic island adjacent to a *pheU* tRNA gene, excises from the chromosome and gets lost at low rate in laboratory growth conditions [13]. In addition, it has been recently described that the prophage-like element φSE14 of *S.* Enteritidis (another *S.* Enteritidis ROD) also excises spontaneously from the chromosome under standard culture conditions [14].

The few reports that have identified specific conditions that promote the excision and transfer of genomic islands have focused on bacteria infecting plants. For instance, the *Pseudomonas syringae* pv. Phaseolicola genomic island 1 (PPHGI-1) excises from the chromosome and is transferred to recipient strains at high rates during the infection of host plants [15]. In addition, the frequency of transfer to different strains of the same species is enhanced *in vitro* when plant apoplastic fluids are added to bacteria undergoing transformation, suggesting that excision and transfer of this genomic island can be influenced by components derived from the host [15]. Also, it has been described that the density of the bacterial population is another factor that influences the excision rate of genomic islands, as it was observed for the symbiotic bacterium *Mesorhizobium loti* [16]. Furthermore, there is one single study in bacteria causing disease in animals describing that the excision of the *Vibrio* Pathogenicity Island 2 from *Vibrio cholerae* can be induced at low temperatures and after UV irradiation [17]. However, the conditions or signals responsible of promoting or preventing the excision of unstable genomic islands in *Salmonella* remain largely unknown. Furthermore, whether the excision of these genetic elements contributes to pathogenicity is an important question that requires to be addressed.

Here we show that ROD21 is an unstable *Salmonella* pathogenicity island that can excise from the bacterial chromosome due to at least two different and independent recombination events. However, only site-specific recombination could lead to ROD21 loss, suggesting that this pathogenicity island may be kept as an episomal element inside the bacterium. Of major importance was the observation that the excision rate of ROD21 increases when *S.* Enteritidis resides inside phagocytic cells, such as dendritic cells and macrophages. These results suggest that the excision frequency of ROD21 can be enhanced by specific environmental conditions taking place inside phagocytic cells during the oxidative stress response against intracellular bacteria.

## Results

### Characterization of ROD21 in the genome of S. Enteritidis

**Figure 1:**
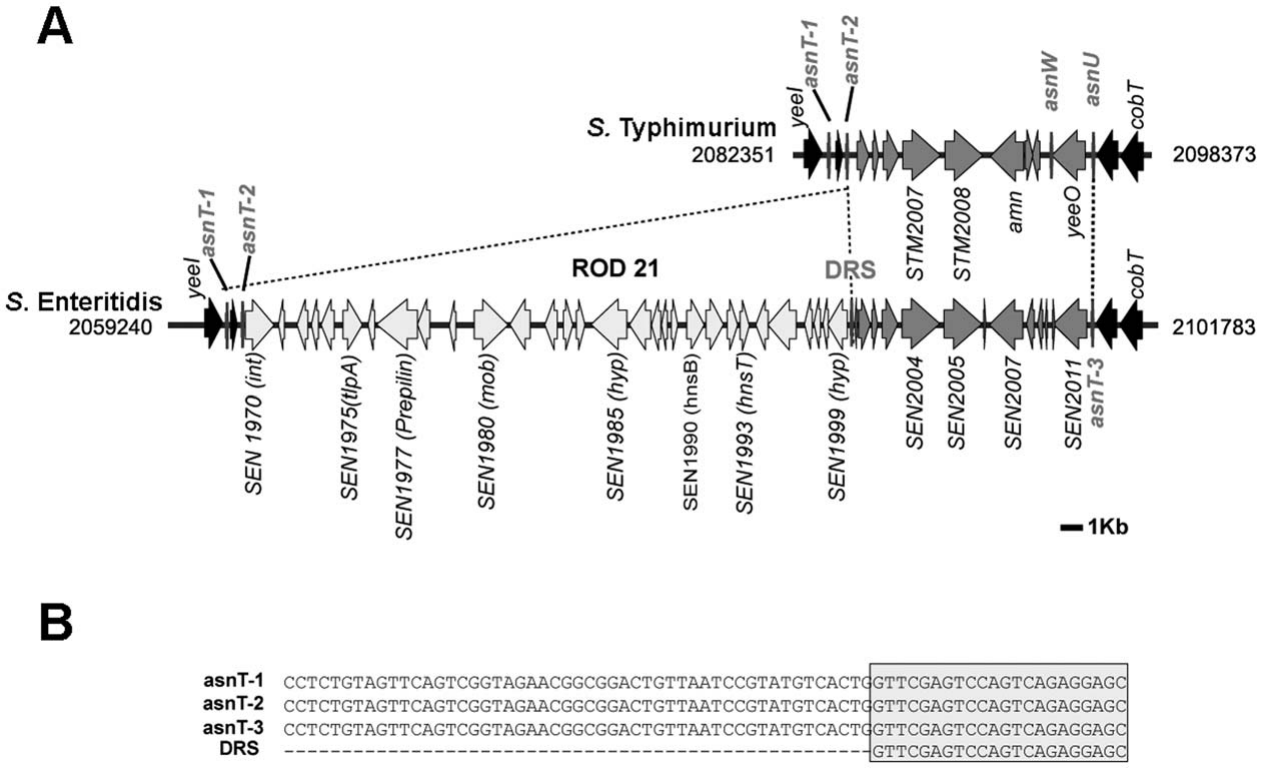
Schematic representation of ROD21 in the chromosome of *S.* Enteritidis. (**A**) Representation of the genetic location of the genes coding for the asparagine tRNA (*asnT-1, -2 and -3*) in the chromosome of *S.* Typhimurium and *S.* Enteritidis and the exact location of ROD21 in the chromosome of *S.* Enteritidis. Black and dark gray arrows represent those genes shared between both serovars and light gray arrows represent genes found only in ROD21 of *S.* Enteritidis. Numbers next to each scheme are coordinates in the chromosome of *S.* Typhimurium and *S.* Enteritidis. DRS stand for Direct Repeated Sequence (*attR*). (**B**) The alignments of DRS and *asnT-1*, *asnT-2* (*attL*) and *asnT-3* show that the DRS is identical to the last 22 bp of the *asnT* genes.

ROD21 is a 26,687 bp DNA fragment located between coordinates 2,061,170 and 2,087,657 in the *S.* Enteritidis PT4 NCTC13349 genome. ROD21 is found in a region of the *S.* Enteritidis chromosome that is common to the genome of *S.* Typhimurium strains LT2 and 14028 (Fig. 1A). ROD21 is found next to an asparagine tRNA gene (*asnT-2* or *attL*) and at its right end is delimited by 24 base pairs (bp), 22 of which are identical to the 3′ end of *asnT* (Fig. 1B). This element was denominated direct repeated sequence (DRS or *attR*). Furthermore, near ROD21 there are other two asparagine tRNA genes located in the same orientation as *asnT-2* (*asnT-1* and *asnT-3* in this study). *asnT-1* is located 971 bp upstream of *asnT-2*, while *asnT-3* is located 11,487 bp downstream of the DRS. A fourth asparagine tRNA gene (*asnW*) is located 9,943 pb downstream of the DRS, but in the opposite direction when compared to the other *asnT* genes.

ROD21 harbors 29 coding sequences that are similar to genes previously described in other bacteria, or that have functions already assigned in *Salmonella* (Table 1). Interestingly, some regions of this pathogenicity island have a genetic organization similar to a genomic island found in Uropathogenic *Escherichia coli* strain CFT073 [18] (Table 1). One of the genes in ROD21 that has an assigned function is *SEN1975* (or *tlpA*), which encodes a virulence protein that has a Toll/Interleukin-1 receptor (TIR) domain [19]. A previous report has described that this protein interferes with the signaling derived from Toll-like receptor engagement and NF-κB activity in epithelial cells. Furthermore, mutant strains of *S.* Enteritidis lacking this gene cause a less severe disease in mice [19]. ROD21 also contains the gene *SEN1993*, encoding a protein homologous to HnsT from uropathogenic *E. coli*, which releases the suppression of virulence genes in these pathogenic bacteria [20]. Another gene found in this pathogenicity island is *SEN1978* that encodes a putative type IV pilin protein. In addition, ROD21 harbors genes coding for proteins belonging to conjugation systems: TraD (*SEN1979*) and MobA/MobL (*SEN1980*) (Fig. 1 and Table 1). Another CDS with assigned function is *SEN1970*, which encodes a putative integrase sharing 74% similarity to the integrase in prophage P4. Based on the presence of *att* sites and the integrase gene found in ROD21, we hypothesized that this pathogenicity island would excise from the *S.* Enteritidis chromosome.

**Table 1:** Open reading frames (ORFs) in ROD21 Pathogenicity island and comparison with *serU* island of the UPEC CFT073.

ORF	Size	Location	Hypothetical role	*serU* island of the UPEC CFT073	% identity
SEN1970	1275	2061363–2062637	Integrase. P4-like integrase.	c2392	25
SEN1971	252	2062709–2062960	Exported protein (S. Dublin) Identities: 100%	-	-
SEN1972	838	2063439–2064276	Pseudogen (putative membrane protein, S. Gallinarum. Identities: 99%)	-	-
SEN1974	609	2064298–2064906	Hypothetical protein (SeD_A2308. S Dublin) Identities:99%	-	-
SEN1975	882	2065272–2066153	TlpA. Cytoplasmic protein with TIR domain (Salmonella sp.) Identities: 100%	c2398	45
SEN1976	2143	2066381–2068523	Pseudogen (putative type IV prepilin protein, S. Gallinarum. Identities: 99%)	c2394	76
SEN1978	558	2068581–2069138	Type IV Pilin (S. Enteritidis). Identities: 100%. (N-terminal PilS domain).	c2395	93
SEN1979	306	2069546–2069851	Conserved hypothetical protein (S. Enteritidis and S. Dublin). Identities: 100%. (Conjugal transfer protein TraD domain)	c2396	87
SEN1980	1521	2070488–2072008	MobA/MobL family protein (S. Dublin) and possible Conjugal transfer protein (S. Enteritidis). Identities: 100%. (MobA/MobL family).	c2397	84
SEN1981	915	2072032–2072946	Conserved hypothetical protein (S. Enteritidis). Identities: 99%	-	-
SEN1981A	264	2073108–2073371	Membrane protein		
SEN1982	543	2073382–2073924	Lipoprotein (S. Enteritidis). Identities: 100%.	c2401	96
SEN1983	488	2074332–2074819	Pseudogen (exported protein. S. Gallinarum. Identities: 99%)	-	-
SEN1984	378	2074852–2075229	Exported protein (S. Enteritidis). Identities: 100%	-	-
SEN1985	1611	2075307–2076917	Hypothetical protein (S. Gallinarum and S. Enteritidis). Identities: 100% (S-adenosylmethionine-dependent methyltransferases (SAM or AdoMet-MTase) domain)	-	-
SEN1986	963	2076969–2077931	Hypothetical protein (S. Dublin and S. Enteritidis). Identities: 100%	c2406	86
SEN1987	423	2077987–2078409	Hypothetical protein (S. Enteritidis). Identities: 100%	-	-
SEN1988	270	2078458–2078727	Hypothetical protein (S. Gallinarum and S. Enteritidis). Identities: 100%	-	-
SEN1989	300	2079026–2079325	Hypothetical protein (S. Enteritidis). Identities: 100%	-	-
SEN1990	735	2080087–2080821	DNA-binding protein (S.Dublin and S. Enteritidis). Identities: 100% (Domain: helix_turn_helix multiple antibiotic resistance protein)	-	-
SEN1991	792	2080852–2081643	Hypothetical protein (S.Gallinarum and S. Enteritidis). Identities: 100%	-	-
SEN1992	480	2081729–2082208	Hypothetical protein (S. Gallinarum). Identities: 98%.	c2410	92
SEN1993	405	2082369–2082773	DNA-binding protein (histone-like protein hlp-II) (S. Gallinarum and S. Enteritidis). Identities: 100% (Domain: global DNA-binding transcriptional dual regulator H-NS; Provisional)	c2411	91
SEN1994	567	2083189–2083755	Membrane protein (S. Gallinarum and S. Enteritidis). Identities: 100%.	-	-
SEN1995	1272	2083802–2085073	Conserved Hypothetical protein (S. Dublin and S. Enteritidis). Identities: 100%.	-	-
SEN1996	297	2085319–2085615	Hypothetical protein (S. Gallinarum and S. Enteritidis). Identities: 100%.	c2414	95
SEN1997	303	2085660–2085962	Hypothetical protein (S. Gallinarum and S. Enteritidis). Identities: 100%.	c2415	95
SEN1998	219	2086032–2086250	Phage regulatory protein (Salmonella sp.). Identities: 100%. (Domain: Prophage CP4-57 regulatory protein (AlpA)/Predicted transcriptional regulator [Transcription])	-	-
SEN1999	876	2086401–2087276	Hypothetical protein (S. Gallinarum and S. Enteritidis). Identities: 100%.	1999	40

### At least two different recombination events promote ROD21 excision from the bacterial chromosome

**Figure 2:**
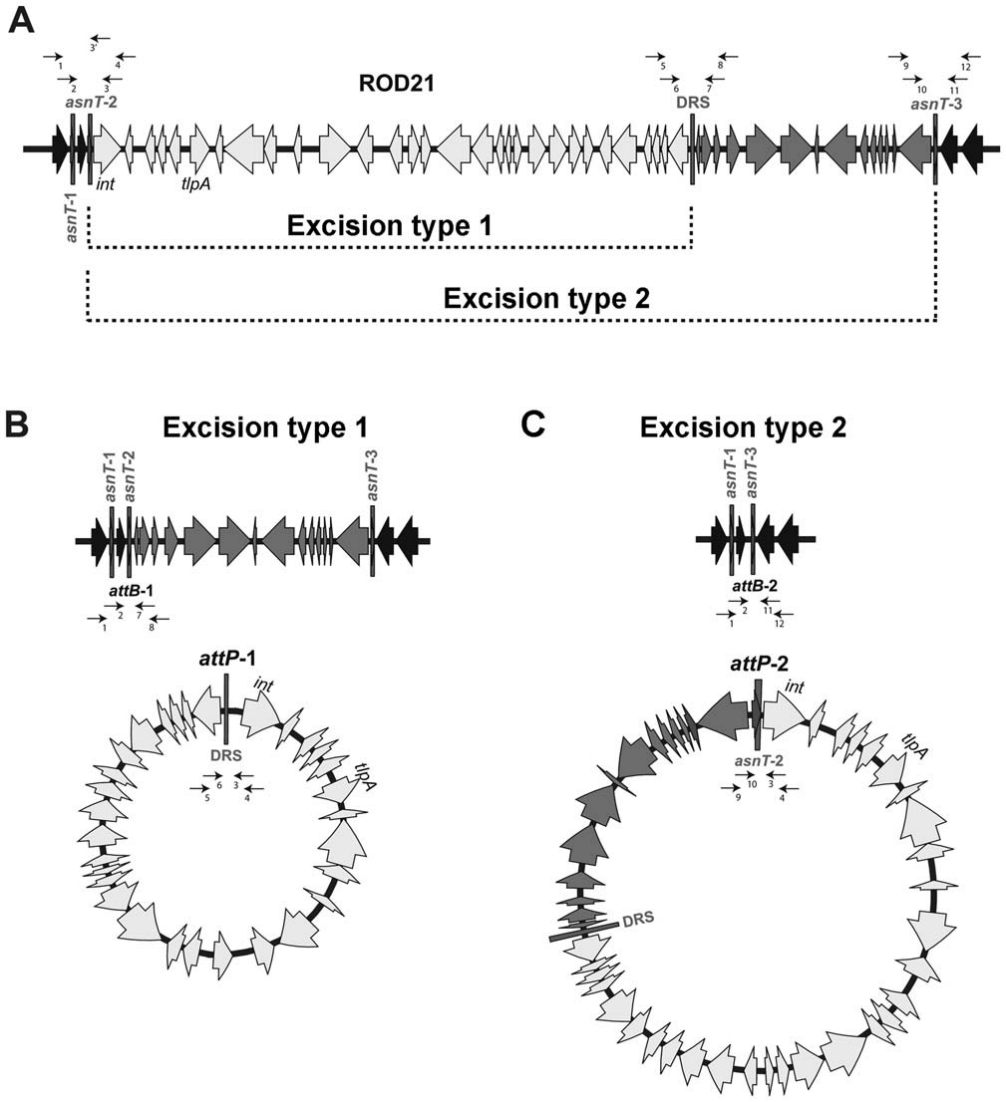
Schematic representation of excisions type 1 and type 2 of ROD21 and the respective episomal elements generated. (**A**) Schematic representation of ROD21 and its surrounding region in the chromosome of the *S.* Enteritidis NCTC13349 strain. Light gray arrows indicate genes that are part of ROD21, black arrows indicate neighboring genes located outside ROD21 and dark gray arrows show neighboring genes specifically contained between the DRS limiting ROD21 and the *asnT-3* gene. Portions of the chromosome involved in type 1 and 2 excisions are shown by connecting the respective recombining DRS/tRNAs (dotted lines). Numbered arrows indicate the regions where the primers used in this study hybridize. (**B**) Schematic representation of the *attB-1* and *attP-1* sites formed after type 1 excision and the genes remaining in both the chromosome of *S.* Enteritidis and the episomal element. (**C**) Schematic representation of the *attB-2* and *attP-2* sites formed after type 2 excision, and the genes remaining in both the chromosome of *S.* Enteritidis and in the episomal element. Primer pairs used to detect the chromosomal excisions and episomal elements are indicated as black arrows.

Next, we tested whether ROD21 is able to undergo spontaneous excision from the bacterial chromosome. Because ROD21 is flanked by three copies of *asnT* and delimited by the DRS, at least two types of recombination events may take place: recombination between *asnT-2* and the DRS (excision type 1) and recombination between *asnT-2* and *asnT-3* (excision type 2). Either one of these recombination events might result in the complete excision of this new pathogenicity island (Fig. 2).

**Figure 3:**
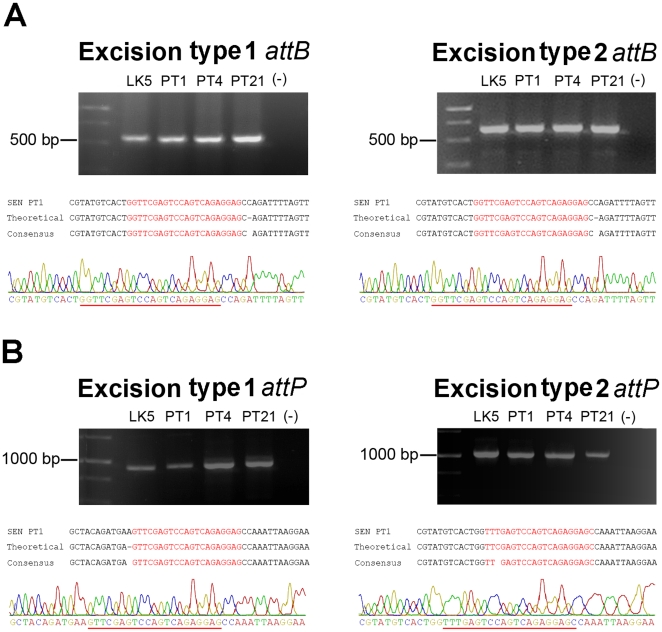
ROD21 excision can be generated by means of two different recombination events. Amplification of *attB* and *attP* sequences generated after type 1 and type 2 excisions were detected by nested PCR in LK5, PT4, PT1 and PT21 strains of *S.* Enteritidis, using primer pairs described in Table 2 and in Figure 2. PCR products for *attB* (**A**) and *attP* (**B**) sequences for each type of excision were resolved in 1% agarose gels. The sequence of each PCR product was obtained (chromatograms in each Figure) and compared with the *attB* and *attP* sequences deduced for type 1 and type 2 excisions (labeled as theoretical). *attB* and *attP* sequences are highlighted in red in both alignments and chromatograms. Expected size for each PCR product: 591 bp for type 1 excision *attB*, 657 bp for type 2 excision *attB*, 995 bp for type 1 excision *attP* and 1050 bp for type 2 excision *attP*.

To evaluate whether these potential recombinations can occur, we performed a PCR reaction using primers that hybridize upstream and downstream of *asnT* genes and the DRS. As shown in Fig. 2B and 2C, these hypothetical excision events yield two different *attB* and *attP* sequences, which could be detected by PCR using several different primer combinations. To detect these excisions, the genomic DNA of four *S.* Enteritidis strains was obtained as described in materials and methods and tested by PCR. Conventional PCR amplifications failed to produce measurable amounts of the expected PCR products for each of the *attB* and *attP* sequences in each of the *S.* Enteritidis strains evaluated (data not shown). To increase the sensitivity of detection, nested PCRs were performed as described in material and methods and the expected sized amplicons were obtained: 591 and 657 bp for the chromosomal *attB-1* and *attB-2* respectively (Fig. 3A) and 958 and 1058 for the episomal *attP-1* and *attP-2* respectively (Fig. 3B). To corroborate the specificity of these PCR products, the DNA fragments obtained from *S.* Enteritidis PT1 were sequenced. As shown in Fig. 3, each of the obtained PCR products matched the expected *attB* (Fig. 3A) and *attP* (Fig. 3B) sequences. These data suggest that both recombination events occurred at low frequency when bacteria grew to stationary phase in LB medium.

### Excision type 2 results in the loss of ROD21 from the chromosome of S. Enteritidis

**Figure 4:**
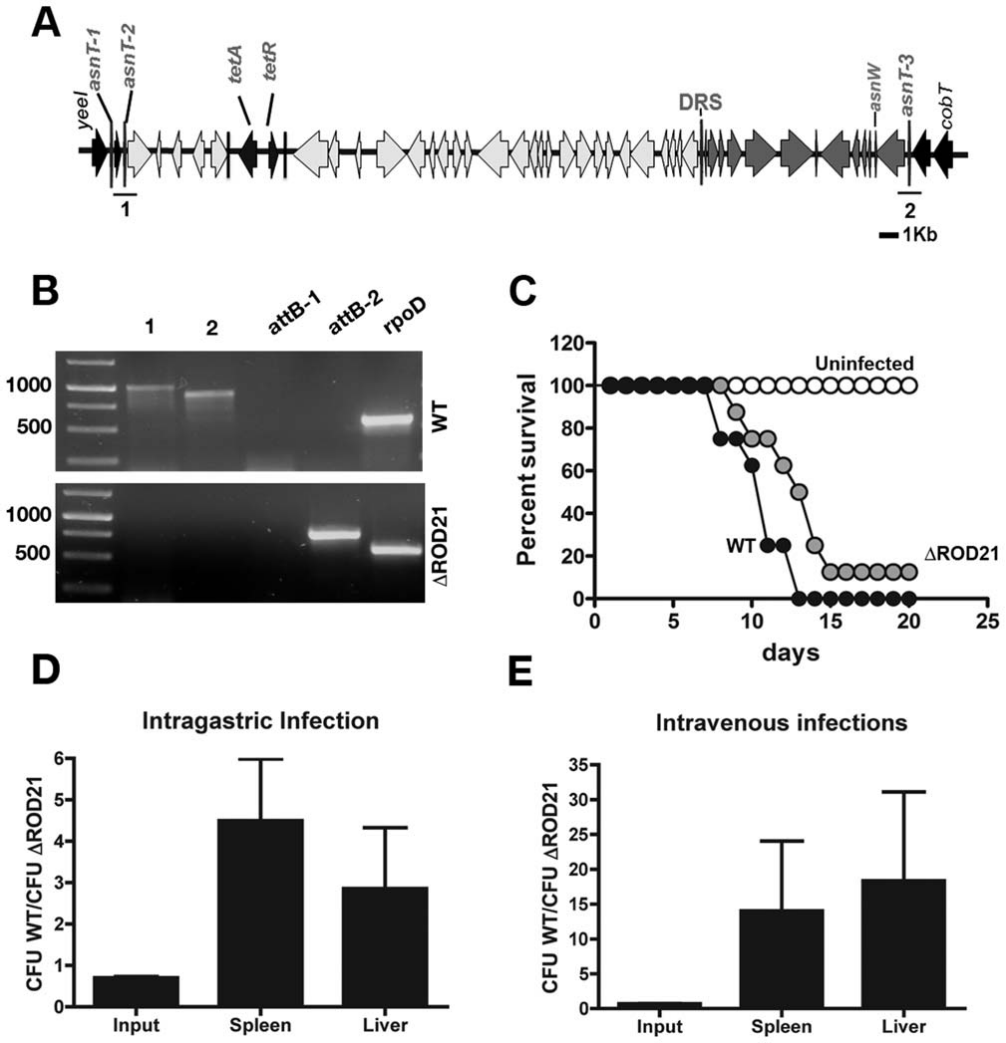
ROD21 is lost in bacteria undergoing type 2 excision. (**A**) Schematic representation of *tetRA* insertion in the ROD21::*tetRA* strain. This strain was used to isolate bacteria that have lost ROD21. The ROD21::*tetRA* strain was grown in the contraselection BM medium and the arising colonies were tested for the presence of ROD21 by PCR. The lines denominated 1 and 2 represent the expected PCR products that would be generated if ROD21 was inserted in the chromosome. (**B**) Detection of ROD21 by PCR analysis in WT *S.* Enteritidis and in one of the ΔROD21 strains isolated in a contraselection assay. This figure shows a representative agarose gel (1%) resolving the PCR products 1 and 2 (which denote each boundary of the integrated form of ROD21) obtained for WT and ΔROD21 *S.* Enteritidis strains. In addition, the *attB* sequences generated after type 1 (*attB-1*) and type 2 (*attB-2*) excisions were also detected by PCR. As a positive control, the *rpoD* gene was amplified by PCR. Data shown derive from one representative *S.* Enteritidis ΔROD21 strain selected out of 6 strains recovered in two independent experiments in which the *attB-2* sequence was detected. (**C**) C57BL/6 mice were orally infected with 1×10^6^ CFUs of either WT or ΔROD21 *S.* Enteritidis strains and the survival rate was measured daily. Uninfected mice were included as controls. Data shown are averages of two independent experiments, each including at least 4 mice per group. (**D and E**) Competitive infection assays, consisting of C57BL/6 mice infected either orally or intravenously with a mixture of the WT (Kn^R^) and ΔROD21 (Cm^R^) *S.* Enteritidis strains (input ratio equal to 1∶1). After 72 h, bacteria were recovered from spleens and livers of infected mice and the ratio of WT (Kn^R^) over ΔROD21 (Cm^R^) *S.* Enteritidis was calculated and compared to the input. Data shown in graphs are average values from two independent experiments for bacteria recovered from spleens and livers after intragastric gavage (**D**) or intravenous (**E**) infections of 3 mice per group.

Then, we evaluated whether the excision of ROD21 results in the loss of this pathogenicity island from the bacterial genome. To evaluate this possibility, we inserted the genes *tetA* and *tetR* downstream the gene *SEN1975* in the strain of *S.* Enteritidis Phagotype 1 (PT1), to generate the ROD21::*tetRA* strain (Fig. 4A). The genes *tetA* and *tetR* confer resistance to tetracycline, but also prevent the growth of tetracycline-resistant bacteria in a medium containing zinc chloride and fusaric acid (BM medium), as described previously [21]. Therefore, only those *Salmonella* strains sensitive to tetracycline will grow in BM medium [21]. Nested PCR reactions showed that excision type 1 and type 2 (Fig. 2A) occurred in the modified ROD21::*tetRA* strain, as efficiently as observed for the wild type (WT) strain (data not shown). To evaluate if these excisions caused ROD21 loss, the ROD21::*tetRA* strain was grown in LB medium and then seeded on solid BM medium, as described in materials and methods. Bacteria were incubated 36 h at 37°C to select for tetracycline sensitive bacteria. A total of 35 tetracycline sensitive colonies out of 137 seeded plates were tested by PCR to evaluate ROD21 loss. We observed that only 6 out of the 35 colonies isolated had lost ROD21 from the chromosome, indicating that the frequency of ROD21 loss is 4.38×10^−8^. Further, PCR reactions showed that only the *attB* sequence generated by excision type 2 could be detected in the genome of all isolated *S.* Enteritidis strains (Fig. 4B), suggesting that excision type 2 was responsible of ROD21 loss in all isolated CFUs. With regard to the other 29 colonies that lost tetracycline resistance without excising ROD21, it is possible that they might had undergone other type of mutations resulting in tetracycline sensitivity, such as mutations in *tetA* or *tetR* genes [22], [23] or changes in cell membrane permeability. However, further research would be required to clarify this issue.

### S. Enteritidis strains that have lost ROD21 show reduced virulence

To evaluate the impact of ROD21 loss in the virulence of *S.* Enteritidis, groups of C57BL/6 mice were orally infected with either WT *S.* Enteritidis or one of the isolated strains of *S.* Enteritidis lacking ROD21 (ΔROD21). After infection, the survival rate of mice was evaluated on a daily basis. As shown in Fig. 4C, mice infected with the ΔROD21 strain survived longer than did mice infected with the WT *S.* Enteritidis strain. However, 15 days after the infection, over 80% of the mice in both groups had died due to *Salmonella* infection. These data suggest that the loss of ROD21 causes an apparent mild reduction of the *S.* Enteritidis virulence in mice. To further study the attenuation of the ΔROD21 strains, we performed in vivo competition assays consisting of either oral or intravenous infection of mice with a mixture of WT and ΔROD21 strains (at a ratio equal to 1). After 72 h of infection, colonizing bacteria were recovered from spleens and livers of infected mice to evaluate the ratio of WT/ΔROD21 in these organs. As shown in Fig. 4D and 4E, the WT strain was recovered in larger proportions in spleens and livers of infected mice 72 h after infection, as compared to the ΔROD21 strain. Because similar data were obtained independently of the route of infection (orally or intravenously), it is likely that the genes encoded by ROD21 might be required for the systemic phase of *Salmonella* infection.

### ROD21 excision is induced during infection of phagocytic cell

To determine whether the rate of ROD21 excision changes during infection, quantitative real time PCR assays were performed to measure the number of bacteria that underwent excision type 1 under different growth conditions. Because an important step in *S.* Enteritidis infective cycle is the invasion and survival in phagocytic cells, ROD21 excision frequency was determined for *S.* Enteritidis while infecting phagocytic cells, such as macrophages and dendritic cells (DCs). Genomic DNA was obtained from *S.* Enteritidis PT1 strain recovered at different steps during a gentamicin protection assay, as described in materials and methods. Then, the copy number of the *attB* sequence for each sample was quantified by using quantitative real time PCR (qPCR). In these assays, the copy number of *attB* sequences represents the amount of bacterial chromosomes that underwent ROD21 excision. qPCR data were normalized based on the total amount of DNA for each sample. Further, the copy number of the *rpoD* gene was quantified as an indication of the total amount of bacterial chromosomes per sample. Data were expressed as the number of *attB* copies/number of *rpoD* copies.

**Figure 5:**
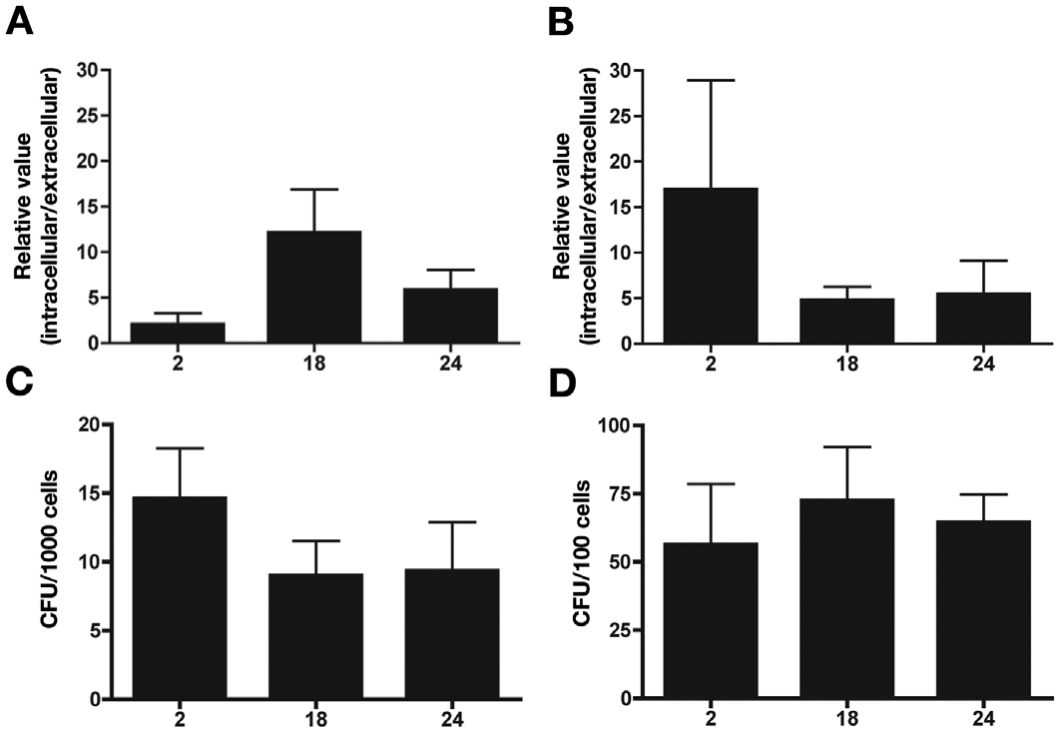
ROD21 excision frequency increases when *S.* Enteritidis infects phagocytic cells. Bone marrow-derived DCs (**A**) and J774.3 macrophages (**B**) were infected with *S.* Enteritidis (MOI equal to 25). After 2, 18 and 24 h post infection (hpi) intracellular bacteria were recovered and the copy number of the *attB* sequence generated by type 1 excision was detected by quantitative PCR, using as template the genomic DNA obtained from intracellular bacteria. Frequency of excision is expressed as the ratio between the copy number of the *attB-1* sequence determined for intracellular and extracellular bacteria. The DNA amount was normalized by calculating the copy number of the *rpoD* gene. Data shown in graphs are average values of at least three independent experiments. The amount of intracellular bacteria recovered after 2, 18 and 24 hpi from DCs (**C**) and J774.3 (**D**) was determined by lysing either 1,000 DCs or 100 J774.3 cells with PBS-triton X100 (0.1%) and seeding the lysates in LB plates. Data shown are the average of at least 3 independent experiments.

Excision type 1 of ROD21 was determined for extracellular bacteria, which were recovered from the supernatant of phagocytic cells 2 h after infection. Further, ROD21 excision was also determined for intracellular bacteria recovered from phagocytic cells at different times post-infection (2, 18 and 24 h). Then, the ratios of the ROD21 excision between intracellular bacteria and extracellular bacteria were determined (relative value in figure 5). As shown in Fig. 5A, excision rates in intracellular bacteria were increased at all time points, especially after 18 h of DCs infection (Fig. 5A) and after 2 h of macrophages infection (Fig. 5B). Similar results were obtained when the ratio of ROD21 excision between intracellular bacteria and bacteria grown in either LB or cell culture media was determined (data not shown). Finally, because similar intracellular bacterial loads were observed at all time points during infection of phagocytic cells (Fig. 5C and 5D), it is unlikely that our results could be due to variability in the amount of bacteria recovered after infection. These findings suggest that the excision of ROD21 might be induced by the environmental conditions found by *S.* Enteritidis inside phagocytic cells during infection.

### Peroxide treatment increases ROD21 excision in vitro

**Figure 6:**
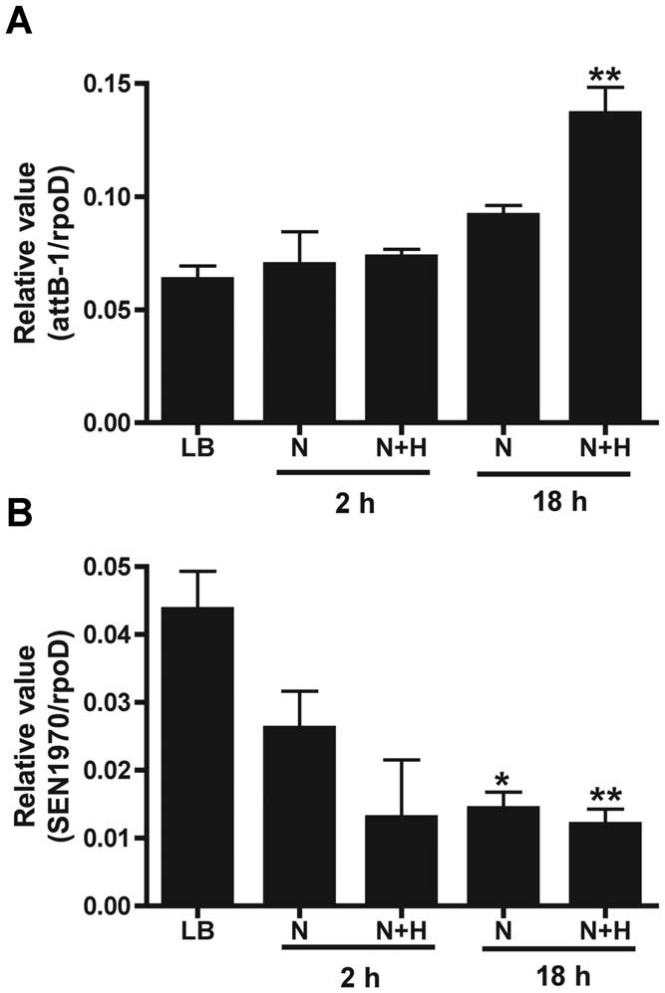
Exposure to peroxide induces the excision of ROD21 from the *S.* Enteritidis chromosome. *S.* Enteritidis strain PT1 was grown in LB medium and then approximately 3×10^9^ CFUs were transferred to fresh LB medium (LB) or to N-minimal medium (N), and incubated for 2 or 18 additional hours. Hydrogen peroxide was added at a final concentration equal to 0.25 mM during the last 30 min of incubation (N+H) and either genomic DNA or RNA was isolated. (**A**) The frequency of *attB-1* excision was quantified by qPCR using genomic DNA and was expressed as a relative value equal to the ratio between the copy number of *attB-1* over the copy number of *rpoD* gene. (**B**) *SEN1970* expression was determined by qPCR using cDNA and expressed as a relative value (the ratio between the copy number of *SEN1970* and the copy number of *rpoD*). The results are the average of three independent experiments. **; <0.01, one-way ANOVA and Tukey post test.

Given that phagocytic cells produce reactive oxygen species upon phagocytosis of bacteria [24], whether oxidative stress could increase the frequency of ROD21 excision was evaluated. *S.* Enteritidis PT1 was grown in LB medium until OD_600_ equal to 0.6 and then 3.6×10^9^ bacteria were incubated in N medium, which mimics the intracellular conditions found inside eukaryotic cells (i.e. reduced magnesium concentration [25]). At 30 min before the end of the incubation in N medium, bacteria were treated with 0.25 mM hydrogen peroxide and the frequency of ROD21 excision was determined by qPCR. As shown in figure 6A, ROD21 excision was significantly higher in bacteria grown for 18 h in N medium and treated with peroxide, as compared to the same strain grown in N medium alone.

To explore a possible mechanism for the enhancement of excision induced by oxidative stress, the relative expression of the gene coding for the integrase (*SEN1970*) required for the integration and excision process was measured. We obtained mRNA from bacteria grown either on LB or N medium that were treated or not with hydrogen peroxide and evaluated the relative amount of the *SEN1970* mRNA, using qPCR. As shown in Fig. 6B, the amount of *SEN1970* mRNA was reduced when bacteria were exposed to N medium and hydrogen peroxide. These results suggest that intracellular conditions, such as reduced nutrients and oxidative stress may increase the excision rate of ROD21 due to a decreased expression of integrase, as it has been described previously for other bacteria [26].

In summary, our data suggest that the intracellular environment of dendritic cells and macrophages promotes excision of ROD21. It is likely that the survival of *Salmonella* inside these phagocytic cells might be increased by the excision of this pathogenicity island.

## Discussion

The acquisition of genes through lateral transfer is a major source of variation and evolution for pathogenic bacteria. An example of mobile elements that can be transferred from one bacterium to another are pathogenicity islands, which can harbor several genes required by bacterial pathogenesis during infection [27]. Previous studies have described that some pathogenicity islands can excise from the bacterial chromosome, either spontaneously or in response to certain stimuli [28]. In this study, we have provided evidence suggesting that ROD21, a pathogenicity island of *S.* Enteritidis, is an unstable genetic element that can undergo spontaneous excision by two different recombination events. Our data show that recombination of *asnT-2* (*attL*) with the DRS (*attR*) results in the excision of ROD21 (excision type 1), which forms a circular episomal genetic element. In addition, recombination of *asnT-2* with *asnT-3* (excision type 2) also leads to the excision of ROD21 from the chromosome, but in this case the excision leads to a circular DNA fragment that includes ROD21 and the fragment located between DRS and *asnT-3*. Both circular elements generated by excisions type 1 and 2 were detected in this study for *S.* Enteritidis. The presence of a gene coding for an integrase (*SEN1970*) in ROD21 suggests that this protein may catalyze excisions type 1 and 2. However, it remains to be determined whether this gene encode a functional protein. Further, it is also possible that other integrases encoded by genes located in different sites in the chromosome could catalyze the excision of this region, as it has been described for pathogenicity islands of *E. coli* [8]. Mechanisms of homologous recombination, known to participate in the excision of unstable genetic elements [29], could also contribute to the excision of ROD21. Given that a gene encoding the RecA protein is present in the *S.* Enteritidis genome [30] and that the sequences involved in the recombination events are 100% identical, it is likely that this protein may also contribute to catalyzing ROD21 excision. Moreover, the presence of three tRNA genes near ROD21, all of them identical in sequence, raises the possibility that 3 additional recombination events could take place: (1) recombination between *asnT-1* and the DRS, (2) recombination between *asnT-1* and *asnT-3*, and (3) recombination between *asnT-3* and the DRS. To define whether these additional excisions occur further research would be required.

Here we have determined that ROD21 excision may result in the loss of this genomic region in a small percentage of the bacterial population, consistently to what has been observed for other bacteria possessing unstable pathogenicity islands [8]. By using a selection assay based on tetracycline sensitivity, bacteria that had lost ROD21 due to type 2 excision (recombination between *asnT-2* and *asnT-3* genes) were isolated. However, bacteria that lost ROD21 due to recombination between *asnT-2* and the DRS could not be isolated, even if both, *attB* and *attP* sequences generated by type 1 excision could be detected by PCR. It is likely that type 1 excision leads to the formation of a stable episomal element, which is kept inside bacteria and probably re-integrated into the chromosome. Although the episomal element formed after an excision by recombination between *asnT-2* and *ansT-3* can be detected by PCR, this element might be unstable and eventually degraded. This possibility could explain as to why only bacteria that lost this genomic region only due to type 2 excision could be isolated.

We also observed that type 2 excision-mediated loss of ROD21 extended the time required by *S.* Enteritidis to cause a lethal disease in mice. Further, *in vivo* competitive assays at equivalent infection times showed that the ΔROD21 strain was less capable of colonizing spleens and livers as compared to WT *S.* Enteritidis. These data are in agreement with a potential role for ROD21 as a cluster for virulence genes. This notion finds additional support on a previous report showing that lack of the ROD21-encoded *SEN1975* gene reduces the capacity of *S.* Enteritidis to cause lethal disease in mice [19]. Moreover, ROD21 also codes for *SEN1993*, which is homologous to HnsT, a protein that promotes the expression of virulence genes in uropathogenic *E. coli*. It is likely then that *SEN1993* could work in *S.* Enteritidis as an inducer of the expression of virulence genes and that the lack of this protein would reduce pathogenicity in mutant strains. However, additional research is required to evaluate whether other ROD21 genes contribute to the virulence of *S.* Enteritidis in mice and other hosts.

We observed that the excision frequency of ROD21 increases when bacteria infect phagocytic cells, such as DCs and macrophages. The highest excision frequencies were obtained for bacteria recovered from the intracellular space of infected cells. It is possible that recognition of signals present inside host cells would lead to the activation of proteins involved in ROD21 excision. As a consequence of excision, transcription of genes contained in these unstable genetic elements could be enhanced, as described for unstable elements found in *Corynebacterium glutamicum* [22]. Increased expression of potential virulence genes contained in ROD21 when *Salmonella* locates inside host cells could be relevant for its ability to survive intracellularly.

The conditions present in the *Salmonella*-containing vacuole, such as low pH, high amounts of reactive oxygen species, nitric oxide, reduced Mg^+2^ concentration, among others, could act as signals that promote production or activation of proteins involved in the excision process. In agreement with this notion, our results show that the presence of hydrogen peroxide increases the frequency of ROD21 excision. Furthermore, the transcription of the integrase gene (*SEN1970*) is reduced when bacteria are grown in N medium and exposed to hydrogen peroxide. These data are in agreement with a recent report showing that Phage 4 integrase expression is downregulated to promote the excision of bacteria unstable genetic elements [26]. Then, it is possible that a reduction of integrase expression could promote ROD21 excision in response to phagocytic cell oxidative burst. It has been shown that oxidative burst starts early in macrophages [23], which is consistent with the early increase of ROD21 excision observed when *S.* Enteritidis infects J774.3 cells.

The excision of ROD21 in *S.* Enteritidis during infection of phagocytic cells is analogous to the observation that prophage excision is induced soon after infection. For instance, contact with pharyngeal epithelial cells promotes the excision of 5 prophages from the genome of group A *Streptococcus* [31]. Such a process induces the production of virulence factors encoded by these prophages. To the best of our knowledge, this is the first report describing the occurrence of the excision phenomenon when bacteria locate inside phagocytic cells, one of the most important stages of the infectious cycle of *Salmonella*. Additional studies are required to further evaluate if the excision of the ROD21 pathogenicity island contributes to the virulence of *S.* Enteritidis.

## Materials and Methods

### Ethics statement

All the procedures performed in this study were revised and approved by the Bioethics and Biosafety Committee of the School of Biological Sciences, Pontificia Universidad Católica de Chile (07/06/2007). All animal work was performed according to the Guide for Care and Use of Laboratory Animals (National Institute of Health, USA) and Institutional guidelines were overseen by a Veterinarian.

### Bacterial strains and growth conditions

*S.* Enteritidis strains used in this study are: *S.* Enteritidis LK5 (provided by Dr. Guido C. Mora, Universidad Andrés Bello, Chile), *S.* Enteritidis phagotype 1 (PT1), phagotype 4 (PT4) and phagotype 21 (PT21) (provided by Mrs. Alda Fernandez from the Public Health Institute of Chile, ISP). These strains were stored at −80°C in LB medium supplemented with 20% glycerol and grown in liquid LB medium at 37°C with aeration and agitation. N-minimal medium was composed of 5 mM KCl, 7.5 mM (NH_4_)_2_SO_4_, 0.5 mM K_2_SO_4_, 1 mM KH_2_PO_4_, 0.1 mM Tris-HCl pH 7.4, 30 µM MgCl_2_, 0.2% glucose, 38 mM glycerol, and 0.1% casaminoacids; pH was adjusted to 5.0. When needed, hydrogen peroxide was added to the N-minimal medium, at a concentration of 0.25 mM. The strain *S.* Enteritidis ROD21::*tetRA* was generated by Lambda Red-mediated recombination, as described by Datsenko and Wanner [32]. Briefly, a PCR product encoding *tetA* and *tetR* genes was generated by PCR amplification from the mini Tn*10* transposon T-POP [33]. The primers used were asnT_tetRA_(H1+P1) (5′ GGA ACT CTC CAT TGG AGA GAA TAC ATA TCA CTT GGG AAA Aaa tca tta agt taa ggt gga 3′) and asnT_tetRA_(H2+P2) (5′ ATG TTT GTG TTT AAA CAT TAT AAT AAA ATT TAA CTT TTA Ata tca aaa tca tta agg tta 3′). The first 40 bp of these primers (capital) align with bp 2,066,174–2,066,213 and 2,066,321–2,066,360 of the *S.* Enteritidis chromosome, respectively. The last 20 bp of these primers (lowercase) align with the T-POP transposon. Competent *S.* Enteritidis PT1 harboring the thermosensitive plasmid pKD46 were prepared as described [32] and PCR products containing *tetA* and *tetR* genes were electrotransformed to these competent cells. After electrotransformation, bacteria were incubated for 1 h at 37°C with aeration in 1 ml of LB medium, and then seeded on solid LB medium supplemented with 25 µg/ml tetracycline. In order to verify the correct insertion of *tetA* and *tetR* genes, a PCR amplification was performed with genomic DNA of the mutant strains and primers SEN_1975_Fw (5′ TTCTGATGAGCAGCGTAAAGAGGC 3′) and asnT_tetRA_(H2+P2). This PCR generates a product of 3,108 bp only if *tetA* and *tetR* genes are inserted in the correct position within ROD21.

### Molecular biology techniques

The genomic DNA used in this work was prepared using the phenol-chloroform method described in [34]. PCR amplification was performed using standard PCR amplification cycles in a MaxiGene Gradient Thermocycler (Axygen). Approximately 1 ng/ml of DNA, 1 nmol/ml of each primer, 0.2 mM deoxynucleoside triphosphates, 1.5 mM MgCl_2_ and 50 U/ml of Taq DNA polymerase (Invitrogen) were used in these amplifications. Nested PCR was performed as described above using 1 µl of a first PCR product as a template. PCR products were resolved by electrophoresis in 1% agarose gels containing 0.5 µg/ml ethidium bromide and visualized under UV light. To determine the specificity of the amplification reaction, some PCR products were gel-purified and cloned into the pCR®-2.1®-TOPO cloning plasmid, according to the manufacturer's instructions (Invitrogen) and sequenced by the Sequencing Facility at the Pontificia Universidad Católica de Chile.

Total RNA was obtained from each bacterial sample using TRIzol reagent (Invitrogen) and purified with RNeasy Mini kit (QIAGEN) according to the manufacturer instructions. After purification, RNA was treated with the DNA Free kit (Ambion) to remove contaminating genomic DNA. DNAse-treated RNA was tested for DNA contamination amplifying *rpoD* gene by PCR. Samples with no positive amplification up to cycle 30 were considered to be DNA free. cDNA synthesis was performed using the ImpromII Reverse Transcription System (Promega) following the manufacturer instructions. One µg of RNA was used as a template and cDNA synthesis was performed using random hexamers. Reactions with no RNA or no reverse transcriptase were included to rule out gDNA contamination.

### Isolation of tetracycline-sensitive S. Enteritidis

*S.* Enteritidis PT1 ROD21::*tetRA* was grown in LB medium supplemented with 25 µg/ml tetracycline at 37°C, with aeration provided by shaking, until an optical density at 600 nm (OD_600_) equal to 2.0 was reached. Serial dilutions were performed in sterile phosphate-buffered saline (PBS) to obtain 100 CFU in a volume of 100 µl. This volume was plated on LB agar supplemented with tetracycline and incubated at 37°C for 18 h. One colony was selected and grown in 2 ml of LB medium without antibiotics at 37°C for 18 h. Serial dilutions of this culture medium were prepared and aliquots of 100 µl containing 10^6^ CFU were plated on solid Bochner-Maloy medium (5 g/l tryptone, 5 g/l yeast extract, 10 g/l NaCl, 10 g/l NaH_2_PO_4_ • H_2_O, 50 mg/l chlortetracycline hydrochloride, 12 mg/l fusaric acid, 0.1 mM ZnCl_2_, 15 g/l agar) [21]. For each experiment, 100 plates were seeded with 1×10^6^ CFU each and the amount of tetracycline sensitive colonies was quantified. In addition, aliquots of 100 µl containing approximately 100 CFU were plated on LB agar to determine the exact CFU seeded in each BM plate. After 36 h of incubation at 37°C, tetracycline-sensitive colonies were selected and grown in liquid BM medium, at 37°C for 4 hours, then patched in LB agar and replica plated in LB agar containing tetracycline and incubated overnight at 37°C. Spontaneous loss of ROD21 in tetracycline-sensitive CFU was confirmed by PCR, using primers pairs indicated in Table 2 and in Fig. 2. The frequency of ROD21 loss was calculated dividing the number of tetracycline-sensitive colonies obtained by the total number of CFU seeded in each experiment.

**Table 2:** Primers used in this study.

Primer	Nucleotide sequence	Coordinates*
1	GACGGAAATCTTTTCGCCTG	2060716-2060735
2	GCGTCAGACTGCCTGTATCA	2060768-2060787
3	CGGCGTAATCTTCTGACCAT	2061726-2061707
3′	TTTGGCGACGACACGGAACGAG	2061683-2061662
4	TAGCGGGATCTCTTCCAGCT	2062007-2061988
5	CAGCAAGACCCTGCCAGAGT	2087049-2087068
6	GCATCATAGCGGCTAACATC	2087203-2087222
7	GGTTAATGCCATAGGAGGGG	2087830-2087811
8	AGTGGGCTTATTGGGATCGG	2087962-2087943
9	TCATAATCACCAGCGACTGG	2098867-2098886
10	CCCAGGCTAAAGGCAACCAC	2098923-2098942
11	CAGGCCTGGCCTTTAAATATCCT	2099721-2099699
12	GTTAATGAGGTGCTGGAGCG	2099774-2099755
SEN1975 Fw	TTCTGATGAGCAGCGTAAAGAGGC	2065343-2065366
SEN1975 Rev	GCAAGGGGACGGACAAAACTATCT	2065807-2065784
asnT_tetRA_(H1+P1)	GGAACTCTCCATTGGAGAGAATACATATCACTTGGGAAAA*AATCATTAAGTTAAGGTGGA* **	2066174-2066213
asnT_tetRA_(H2+P2)	ATGTTTGTGTTTAAACATTATAATAAAATTTAACTTTTAA*TATCAAAATCATTAAGGTTA* **	2066360-2066321
SEN_1075_Fw	TTCTGATGAGCAGCGTAAAGAGGC	2065343-2065366
rpoD-Fw-2	TGAGTCTGAAATCGGTCGTACG	3264619-3264640
rpoD-Rev	TTCGCGGGTAACATCGAACT	3266113-3266094
rpoD-RT-Fw	GTTGACCCGGGAAGGCGAAA	3264688-3264707
rpoD-RT-Rev	CAGAACCGACGTGAGTTGCG	3264908-3264889
SEN1970-RT-Fw	CGATACTGTCTGGAAGCGCCT	2061725-2061745
SEN1970-RT-Rev	GGACAGCGCCTTCCATATCAT	2061954-2061934
attB1nested-RT-Fw	GTTACTATGCGCCCCGTTCACAC	2061082-2061104
attB1nested-RT-Rev	CCGATTAAGCCCCAAAAACTATG	2087781-2087759

*Coordinates are those of the *S. enterica* serovar Enteritidis PT4 NTCT NCTC13349 sequence.

**Italics indicate the region that anneals to the 5′ or 3′ end of a mini *Tn*10 transposon.

### Mouse infection assays

Groups of 4 male C57BL/6 mice (5–6 week age) were used to evaluate the virulence of strain *S.* Enteritidis PT1 ΔROD21. Infections with WT or ΔROD21 *S.* Enteritidis PT1 strains were performed by growing these bacteria in LB medium at 37°C until an OD_600_ equal to 0.6 was reached. The volume of bacterial culture containing 1×10^6^ CFUs was centrifuged in a refrigerated microcentrifuge (CT15RE Hitachi) at 10,000× g for 5 min. Bacterial pellets were thoroughly resuspended in 20 µl of PBS and used to orally infect mice. Infective doses were corroborated by seeding serial dilutions of the bacterial inoculum onto LB plates. After infection, survival rate was recorded on a daily basis. To perform competitive assays, a kanamycin or chloramphenicol resistance gene was introduced between genes *putA* (*SEN0986A*) and *putP* (*SEN0987*) in WT and ΔROD21 *S.* Enteritidis, respectively, using the methodology described by Datsenko and Wanner [32]. These bacteria were grown until OD_600_ equal to 0.6 was reached and 1×10^6^ CFU of each strain was mixed, resuspended in 200 µl PBS and used to infect groups of C57BL/6 mice, by intragastric gavage. To perform intravenous infection, 1,000 CFU of each strain were mixed, resuspended in 20 µl PBS and injected in the lateral tail vein using an insulin syringe (G29 needle). The initial proportion of kanamycin and chloramphenicol resistant bacteria was determined by seeding bacteria in plates of solid LB, LB/kanamycin (50 µg/ml) and LB/chloramphenicol (10 µg/ml) that were incubated for 12–16 h at 37°C. After 72 h of infection, mice were euthanized and livers and spleens were recovered and homogenized with two slides in a Petri dish, with 2 ml PBS. To determine the total amount of both kanamycin and chloramphenicol resistant bacteria, serial dilutions of the homogenized tissue were seeded in solid LB, LB/Kanamycin (50 µg/ml) and LB/Chloramphenicol (10 µg/ml) medium and incubated for 12–16 h at 37°C. Then, colonies were counted and the competitive index was calculated as follows: (CFU_WT_/CFU_ΔROD21_) *Input*/(CFU_WT_/CFU_ΔROD21_) *Output*. The word “input” refers to the proportion of bacteria used to infect mice and “output” to the bacteria recovered from organs.

### Phagocytic cells assays

The monocyte/macrophage J774.3 cell line used in this study was kindly provided by Dr. María Inés Becker (Biosonda S.A., Chile). J774.3 cells were routinely grown in high-glucose DMEM medium (GIBCO, Invitrogen), supplemented with 10% Fetal Bovine Serum (HyClone) and 1 mM HEPES (GIBCO, Invitrogen) in T75 bottles. Cells were incubated at 37°C and 5% CO_2_ until 95% of confluence. Before infection assays, cells were treated with 0.1 mg/ml trypsine (HyClone) for 5 min, recovered in 50 ml polypropylene tubes, and centrifuged at 1,800× g for 5 min at room temperature. After three washes with supplemented DMEM medium, cell number and viability was determined in a haemocytometer, using the trypan blue staining (1 mg/ml, Invitrogen). 5×10^5^ cells/ml were seeded in 24 well-plates and incubated overnight at 37°C and 5% CO_2_. DCs were prepared from bone marrow precursors of C57BL/6 mice. Cells were incubated in complete RPMI 1640 medium supplemented with 5% FCS (Hyclone), 2 mM glutamine, 1 mM non-essential amino acids, 1 mM pyruvate, 1 mM HEPES, and 10 ng/ml of recombinant murine GM-CSF (Peprotech). All cell culture media were acquired from GIBCO (Invitrogen). Culture media was replaced every 2 days. After 6 days, the phenotype of DCs was analyzed by flow cytometry for the expression of the surface markers CD11c, CD86 and CD40, which revealed over 70% CD11c^+^ with an immature phenotype. Before the infection assays, DCs were washed three times with PBS and then culture media was replaced with complete RPMI medium without antibiotics. DCs and macrophages were infected with *S.* Enteritidis PT1 at a multiplicity of infection (MOI) equal to 25. The MOI was confirmed by plating serial dilutions of bacterial cultures on LB agar. After 1 h of incubation at 37°C and 5% CO_2_, the supernatant of infected cells was recovered and stored for genomic DNA preparation. Cells were washed two times with PBS and 1 ml of appropriate medium supplemented with gentamicin 50 mg/ml was added to cell cultures to kill the remaining extracellular bacteria. After 2, 18 and 24 h of infection, cells were removed from the wells and centrifuged at 783× g for 5 min. No significant changes on cell viability were observed after infection with *S.* Enteritidis at the time points used on the experiments (data not shown). To recover intracellular bacteria, phagocytic cells were treated with 1 ml of lysis solution (19% ethanol, 0.1% SDS, 1% saturated basic phenol) for 30 min on ice. After the incubation period, the cell lysate was centrifuged at 7,043× g for 5 min, and genomic DNA was extracted following the methodology mentioned above. In order to quantify the amount of intracellular bacteria at different time points, either 100,000 DCs or 10,000 J774.3 cells were treated with 1 ml of PBS-0.1% triton X-100 for 15 min at room temperature. 100 µl of cell lysates were plated on LB agar and the plates were incubated at 37°C for 18 h. In parallel, an equal amount of bacteria used to infect cells was incubated in 1 ml of cell medium. The incubation was performed for 2 h and after that time bacteria were recovered by centrifugation at 7.043× g for 5 min, and genomic DNA was prepared as described above.

### Peroxide treatment

To evaluate whether oxidative stress has a role in the excision of ROD21, we used hydrogen peroxide (H_2_O_2_) as an oxidative agent and analyzed its effects in bacterial cultures grown in LB, N-minimal medium, and N-minimal medium+H_2_O_2_. *S.* Enteritidis strain PT1 was grown in LB until OD_600_ equal to 0.6 was reached. Then, the culture was split in 6 samples, each containing approximately 3×10^9^ bacteria. The samples were pelleted and then two of them were resuspended in fresh LB medium while the other four were resuspended in N medium. From these 6 new cultures, two of the N cultures were incubated for an additional 1.5 hour. Next, 0.25 mM H_2_O_2_ (Merck) was added and cultures were incubated for additional 30 min. The other two N samples were incubated for 17.5 hours, then 0.25 mM H_2_O_2_ (Merck) was added and cultures were incubated for additional 30 minutes. After the H_2_O_2_ treatment, all samples were pelleted and stored at −80°C until analyzed. Genomic DNA or total RNA was prepared as described above.

### Quantitative real time PCR assays

Quantitative real time PCR for the quantification of ROD21 excisions was performed using Brilliant SYBR Green QPCR kit (STRATAGENE), following the manufacturer's instructions. The reaction mixture contained 2 µl of genomic DNA as a template and 0.12 pmol/µl of each primer. Standard curves for *attB*-1 and *rpoD* were generated using serial dilutions of a plasmid containing the corresponding PCR fragment for *attB-1* and *rpoD*. Thermal cycling conditions were: segment 1; one cycle at 50°C for 2 min followed by initial denaturation at 95°C for 10 min; segment 2, 40 cycles of 30 s at 95°C, 1 min at 58°C, 1 min at 72°C and 15 s at 80°C; segment 3, 1 cycle of 1 min at 95°C, 30 s at 55°C and 30 s at 95°C. The copy number of *attB-1* and *rpoD* was calculated as follows: The number of *attB-1* and *rpoD* copies was determined by the ratio between the amount of DNA in a sample and the weight of one molecule of the plasmid with the insert. To graph the standard curve, the value of the threshold cycle (Ct) was confronted with the log10 of the initial copy number of each sample, generating a linear relationship that allows us to know the number of copies of the sample, which has a specific Ct [35]. The results were expressed as the ratio of *attB-1* copy number/*rpoD* copy number (relative value).

Quantitative real time for the quantification of *SEN1970* expression was performed as described above, but using as a template 2 µl of a 10^−1^ dilution of the RT-PCR reaction. *rpoD* were used as reference gene to normalize the copy numbers of *SEN1970*. Standard curves were made of serial dilutions of purified PCR products.

### Statistic analyses

Statistics significance was determined using the analyses of variance (ANOVA) test with the Prism Graphpad software.

## References

[1] Guard-Petter J (2001). The chicken, the egg and *Salmonella* Enteritidis.. Environ Microbiol.

[2] Thomson NR, Clayton DJ, Windhorst D, Vernikos G, Davidson S (2008). Comparative genome analysis of *Salmonella* Enteritidis PT4 and *Salmonella Gallinarum* 287/91 provides insights into evolutionary and host adaptation pathways.. Genome Res.

[3] Cotter PA, DiRita VJ (2000). Bacterial virulence gene regulation: an evolutionary perspective.. Annu Rev Microbiol.

[4] Bueno SM, González PA, Carreño LJ, Tobar JA, Mora GC (2008). The capacity of *Salmonella* to survive inside dendritic cells and prevent antigen presentation to T cells is host specific.. Immunology.

[5] Gantois I, Ducatelle R, Pasmans F, Haesebrouck F, Gast R (2009). Mechanisms of egg contamination by *Salmonella* Enteritidis.. FEMS Microbiol Rev.

[6] Porwollik S, Santiviago CA, Cheng P, Florea L, Jackson S (2005). Differences in gene content between *Salmonella enterica* serovar Enteritidis isolates and comparison to closely related serovars Gallinarum and Dublin.. J Bacteriol.

[7] Middendorf B, Hochnut B, Leipold K, Dobrindt U, Blum-Oehler G (2004). Instability of pathogenicity islands in uropathogenic *Escherichia coli* 536.. J Bacteriol.

[8] Hochhut B, Wilde C, Balling G, Middendorf B, Dobrindt U (2006). Role of pathogenicity island-associated integrases in the genome plasticity of uropathogenic *Escherichia coli* strain 536.. Mol Microbiol.

[9] Murphy RA, Boyd EF (2008). Three pathogenicity islands of *Vibrio cholerae* can excise from the chromosome and form circular intermediates.. J Bacteriol.

[10] Turner SA, Luck SN, Sakellaris H, Rajakumar K, Adler B (2001). Nested deletions of the SRL pathogenicity island of *Shigella flexneri* 2a.. J Bacteriol.

[11] Lesic B, Bach S, Ghigo JM, Dobrindt U, Hacker J (2004). Excision of the high-pathogenicity island of *Yersinia pseudotuberculosis* requires the combined actions of its cognate integrase and Hef, a new recombination directionality factor.. Mol Microbiol.

[12] Bach S, Buchrieser C, Prentice M, Guiyoule A, Msadek T (1999). The high-pathogenicity island of *Yersinia enterocolitica* Ye8081 undergoes low-frequency deletion but not precise excision, suggesting recent stabilization in the genome.. Infect Immun.

[13] Bueno SM, Santiviago CA, Murillo AA, Fuentes JA, Trombert AN (2004). Precise excision of the large pathogenicity island, SPI7, in *Salmonella enterica* serovar Typhi.. J Bacteriol.

[14] Santiviago CA, Blondel CJ, Quezada CP, Silva CA, Tobar PM (2010). Spontaneous excision of the *Salmonella enterica* serovar Enteritidis-specific defective prophage-like element phiSE14.. J Bacteriol.

[15] Lovell HC, Mansfield JW, Godfrey SA, Jackson RW, Hancock JT (2009). Bacterial evolution by genomic island transfer occurs via DNA transformation in planta.. Curr Biol.

[16] Ramsay JP, Sullivan JT, Jambari N, Ortori CA, Heeb S (2009). A LuxRI-family regulatory system controls excision and transfer of the *Mesorhizobium loti* strain R7A symbiosis island by activating expression of two conserved hypothetical genes.. Mol Microbiol.

[17] Almagro-Moreno S, Napolitano MG, Boyd EF (2010). Excision dynamics of *Vibrio* pathogenicity island-2 from *Vibrio cholerae*: role of a recombination directionality factor VefA.. BMC Microbiol.

[18] Williamson HS, Free A (2005). A truncated H-NS-like protein from enteropathogenic *Escherichia coli* acts as an H-NS antagonist.. Mol Microbiol.

[19] Newman RM, Salunkhe P, Godzik A, Reed JC (2006). Identification and characterization of a novel bacterial virulence factor that shares homology with mammalian Toll/interleukin-1 receptor family proteins.. Infect Immun.

[20] Thomson N, Baker S, Pickard D, Fookes M, Anjum M (2004). The role of prophage-like elements in the diversity of *Salmonella enterica* serovars.. J Mol Biol.

[21] Maloy SR, WD Nunn (1981). Selection for loss of tetracycline resistance by *Escherichia coli*.. J Bacteriol.

[22] Moyed HS, Bertrand KP (1983). Mutations in multicopy Tn10 tet plasmids that confer resistance to inhibitory effects of inducers of tet gene expression.. J Bacteriol.

[23] McMurry LM, Stephan M, Levy SB (1992). Decreased function of the class B tetracycline efflux protein Tet with mutations at aspartate 15, a putative intramembrane residue.. J Bacteriol.

[24] Araya DV, Quiroz TS, Tobar HE, Lizana RJ, Quezada CP (2010). Deletion of a prophage-like element causes attenuation of *Salmonella enterica* serovar Enteritidis and promotes protective immunity.. Vaccine.

[25] Deiwick J, Nikolaus T, Erdogan S, Hensel M (1999). Environmental regulation of *Salmonella* pathogenicity island 2 gene expression.. Mol Microbiol.

[26] Moyed HS, Nguyen TT, Bertrand KP (2009). Multicopy Tn10 tet plasmids confer sensitivity to induction of tet gene expression.. J Bacteriol.

[27] Juhas M, van der Meer JR, Gaillard M, Harding RM, Hood DW (2009). Genomic islands: tools of bacterial horizontal gene transfer and evolution.. FEMS Microbiol Rev.

[28] Manson JM, Gilmore MS (2006). Pathogenicity island integrase cross-talk: a potential new tool for virulence modulation.. Mol Microbiol.

[29] Lesic B, Carniel E (2005). Horizontal transfer of the high-pathogenicity island of *Yersinia pseudotuberculosis*.. J Bacteriol.

[30] Kobayashi H, Miyamoto T, Hashimoto Y, Kiriki M, Motomatsu A (2005). Identification of factors involved in recovery of heat-injured *Salmonella* Enteritidis.. J Food Prot.

[31] Banks DJ, Lei B, Musser JM (2003). Prophage induction and expression of prophage-encoded virulence factors in group A *Streptococcus* serotype M3 strain MGAS315.. Infect Immun.

[32] Datsenko KA, Wanner BL (2000). One-step inactivation of chromosomal genes in *Escherichia coli* K-12 using PCR products.. Proc Natl Acad Sci U S A.

[33] Hidalgo AA, Trombert AN, Castro-Alonso JC, Santiviago CA, Tesser BR (2004). Insertions of mini-Tn10 transposon T-POP in *Salmonella enterica sv. typhi*.. Genetics.

[34] Sambrook J, Fritsch EF, Maniatis T (1982). Molecular cloning: a laboratory manual.

[35] Higuchi R, Fockler C, Dollinger G, Watson R (1993). Kinetic PCR analysis: real-time monitoring of DNA amplification reactions.. Biotechnology (NY).

